# Post-pandemic self-reported mental health of mental healthcare professionals in the Netherlands compared to during the pandemic – an online longitudinal follow-up study

**DOI:** 10.3389/fpubh.2023.1221427

**Published:** 2023-07-03

**Authors:** Lars de Vroege, Anneloes van den Broek

**Affiliations:** ^1^Tranzo Department, Tilburg School of Social and Behavioral Sciences, Tilburg University, Tilburg, Netherlands; ^2^GGz Breburg, Breda, Netherlands; ^3^Department of Post Academic Psychology Training and Education, Breburg Academy, GGz Breburg, Tilburg, Netherlands

**Keywords:** COVID-19, pandemic, mental health, HCW, mental healthcare workers, resilience, the Netherlands

## Abstract

The mental health of professionals was under pressure during- and post-pandemic. Initially, the focus was mainly on the health workers in the hospitals, but over time the pressure shifted to other sectors, including mental health care. An increase in workload and decrease in mental health of healthcare professionals in mental health care can lead to a decrease in the available care capacity. In an earlier online survey of mental health professionals, 1,300 professionals from a large number of mental healthcare institutions were involved. In this study, conducted in September 2021, about half of the respondents reported increased levels of stress. Feelings of anxiety, anger, and sadness were also increasingly experienced due to the COVID-19 pandemic. Furthermore, 4.2% replied that they were considering resigning their jobs. One of the recommendations of this previous study was to monitor these professionals repeatedly to be able to make an estimate of the stress and vision of work during the course of the pandemic and afterwards. Following this recommendation, the online survey was repeated. The aim of the current online longitudinal follow-up study was to re-evaluated mental status of healthcare workers. 510 healthcare workers participated in this follow-up survey. The reported mental health complaints were significantly higher during compared to post-pandemic. Respondents were less able to maintain work/life balance during the pandemic and even reported a shift to work. However, the majority of respondents indicated that they had restored this balance post-pandemic. Moreover, more sick leave was reported post-pandemic than during the pandemic and more frequent absences post-pandemic. This highlights the importance of focusing on resilience over training and career.

## 1. Introduction

In December 2019 the outbreak of the Coronavirus started in Wuhan city, Hubei Province, China, with a few pneumonia cases. Initially, the virus was found in a few people in Europe and the United States. In March 2020, the World Health Organization (WHO) declared the COVID-19 outbreak a pandemic (1) and varying periods of lockdown and social distancing followed with falling infection rates and new lockdown periods as well as an increase in social contradictions (2, 3). Many European countries struggled with overcrowded hospitals followed by a decrease of infections in the summer and an increase of infections in the autumn of 2020 which led to new periods of lockdown. After the first COVID-19 vaccination was carried out in the United Kingdom in December 2020, the start of a global immunization program began and brought hope and perspective for recovery (4, 5). Primarily all attention was paid to the healthcare workers (HCW) at the front in the hospitals given the high pressure they are working on. After a decrease in COVID-19 infections and the related number of hospital admissions, HCW were confronted with the ‘delayed care’ in hospital which caused renewed pressure. However this pressure shifted to other sectors. During the (post)-pandemic period the demand for mental health services continued to increase (6) while Mental Health Care Workers (MHCW) were struggling to provide needed care (7).

In the Netherlands, the DFY-study (Do not Forget Yourself-study) (8) which focusses on mental health of mental healthcare workers reported several results from the first measurement in January 2022 (9). Furthermore, 50% of the employees in mental health care institutions in the Netherlands reported elevated levels of stress and 30% had signs of depression (10) using a self-report questionnaire. An increase in registration at the mental health care institutions took place simultaneously with mental complaints as a result of the COVID-19 pandemic. This increased workload even more, next to the pressure of the lengthening of waiting time for admission. The shift from face to face to telehealth and the confrontation with social differences among the clients and as a consequence conspiracy thinking in the treatment room brought unknown topics of conversation and made a big appeal to the mental healthcare worker (3). Other studies amongst HCW collaborate these results and reported mood and sleep disturbances (11) and post-traumatic stress symptoms (12, 13). Besides these symptoms, also higher levels of psychological distress (14) were reported as a result of the pandemic in another study. These results corroborate the need for preventing MHCW to develop mental health problems and evaluate how to support MHCW.

Based on the findings of a recent scoping review (15) preventing MHCW from mental health problems and maintain sustainable employment during pandemic waves, it is pivotal to use a systematic mental health tool frequently. Screening MHCW with regards to mental health problems provides early protection and realizes the opportunity to signalize future problems and prevent MHCW from sick leave and/or even resigning. The DFY-study (Do not Forget Yourself-study) intends to provide some signals to the government by frequently monitoring of mental health status of MHCW.

This current study reports about the comparison of two groups of MHCW on the experience of mental health related to the workplace and challenges. The first during a period of uncertainty (lockdown and unknown course of COVID-19 in the autumn of 2021) and the second post-pandemic in the autumn of 2022. A repeated measurement design was used to focus on MHCW and following objectives: (i) to assess the experience of increased symptoms of anxiety, sadness, levels of stress, sadness, and anger over time, (ii) to identify challenges regarding work/private life balance, (iii) exploring experience of sick leave, taken days off, and absenteeism, and (iv) exploring considerations about re-organizing work (e.g., working less hours, quitting their job). We hypothesize that levels of mental symptoms decrease over time but the amount of mental health symptoms remains high. Also we hypothesize that some signs of resilience are reflected in the results of our study (e.g., restore of balance between private life and work).

## 2. Materials and methods

### 2.1. Study design

In order to compare the results of the previous survey regarding mental health amongst health care professionals (10), we repeated this survey in the same way. We used social media (LinkedIn) and contact within (large) mental health institutions in the Netherlands to distribute the questionnaire online, using Qualtrics using LinkedIn platform and emailing several mental health care institutions situated in the Netherlands with a personal invitation to participate in the study. Hence the character of this study, internet access was an inclusion criterion, as well as Dutch reading and writing capability. Furthermore, respondents had to be employed within mental health care. The questionnaire was finished in less than 15 min by all participants. Because of the distribution of the internet survey, we did not know how many participants we invited with our request to complete the questionnaire. Therefore, we are unfortunately unable to provide response rates. All participants that started the questionnaire completed the assessment.

We aimed to reach any employee within mental health institutions so all employees that responded were considered ‘professional’ in this article. For the current study we compare two measurements, a recent measure (October–December 2022) versus a previous measure (August–September 2021) referred to as during-pandemic sample and post-pandemic sample, respectively (in the Netherlands, October–December 2022 was declared post-pandemic and all safety measures were discontinued in this period). Our scientific board approved of repeating the current survey in light of the previous survey (CWO 2021–35).

### 2.2. Survey questions

The current survey was identical to the questions in previous survey (10). These questions consisted of questions regarding mental health status regarding symptoms such as sadness, anger, anxiety, and levels of stress. Three questions regarding work/private life balance were included to explore the ability to balance life working from home. Furthermore, three questions were asked related to absenteeism (sick leave, taken days off, and absenteeism). Lastly, we explored whether the respondents were considering working less hours or quitting their job (i.e., re-organizing their work). In order to obtain as much responses as possible, we anonymously obtained all responses so did not ask for sociodemographic variables. Between 13th of October 2022 till the 4th of December 2022, 503 responses were registered. 2 (0.4%) respondents did not start the survey; hence the total sample existed of 510 respondents.

### 2.3. Statistical analyses

First, mental health symptoms and work-related matters based on the post-pandemic survey will be described. Second, reported mental health symptoms and work-related outcomes between the two samples were compared. Categorical variables were presented by means of frequency tables for both samples (pre- and post-pandemic). We explored distribution differences between categorical variables with Chi-Square test (for trend) and used Cramer’s V as effect size. Analyses were conducted using the Statistical Package for the Social Sciences (SPSS) version 27 with an alpha level of 0.05.

## 3. Results

### 3.1. Mental health symptoms post-pandemic

Table 1 shows the results of mental health symptoms and balance work/private and Table 2 shows the results of work-related questions regarding absenteeism and re-organization of work. In general, most respondents stated that they did not experience more/not less symptoms of anxiety, depression, stress, sadness and/or anger. Nevertheless, 35.7% of the respondents (*n* = 182) reported *“more symptoms”* of stress and 20.6% (*n* = 105) of the respondents experienced more symptoms of depression compared to during the pandemic. Between 5.1 and 6.9% of the respondents experienced less mental health symptoms compared to during the pandemic. Row 11–19 of Table 1 describes the results regarding questions focusing on working from home and balancing work and private. 7.5% (*n* = 38) to 22.9% (*n* = 117) stated that *“the balance between work and private tipped towards work due to working from home,”* respectively. 10.2% (*n* = 52) to 29.2% (*n* = 149) reported that they were less able to *“effectively balance work and private.”* Finally, 2.4% (*n* = 12) to 17.3% (*n* = 88) were not able to *“balance work and private (anymore).”*

**Table 1:** Mental health symptoms and balance work and private reported post-pandemic (*N* = 510).

Questions						
Compared to before the COVID-19 pandemic, I experience						
	More symptoms of *n* (%)		Not more/not less symptoms of *n* (%)		Less symptoms of *n* (%)	Missing *n* (%)
Anxiety	51 (10.0)		416 (81.6)		35 (6.9)	8 (1.6)
Depression	105 (20.6)		366 (71.8)		28 (5.5)	11 (2.2)
Higher levels of stress	182 (35.7)		294 (57.6)		29 (5.7)	5 (1.0)
Sadness	49 (9.6)		425 (83.3)		26 (5.1)	10 (2.0)
Anger	96 (18.8)		379 (74.3)		28 (5.5)	7 (1.4)
Compared to before the COVID-19 pandemic:						
Due to working from home, work and private balance has tipped the scale towards work						
	Agree *n* (%)	Partly agree *n* (%)	Neutral *n* (%)	Partly disagree *n* (%)	Disagree *n* (%)	Missing *n* (%)
	38 (7.5)	117 (22.9)	123 (24.1)	72 (14.1)	154 (30.2)	6 (1.2)
I can less effectively balance work and private						
	Agree *n* (%)	Partly agree *n* (%)	Neutral *n* (%)	Partly disagree *n* (%)	Disagree *n* (%)	
	52 (10.2)	149 (29.2)	107 (21.0)	58 (11.4)	139 (27.3)	5 (1.0)
I cannot balance (anymore) between work and private						
	Agree *n* (%)	Partly agree *n* (%)	Neutral *n* (%)	Partly disagree *n* (%)	Disagree *n* (%)	
	12 (2.4)	88 (17.3)	122 (23.9)	93 (18.2)	190 (37.3)	5 (1.0)

Missing values are displayed in last column and reflect missed responses on the according question and are calculated using the total sample of respondents (*N* = 510).

**Table 2:** Work-related questions regarding absenteeism, sick leave, and reorganization of work.

Questions – Which statement holds true regarding sick leave and/or absenteeism during the COVID-19 pandemic?		
	I have taken more sick leave *n* (%)	Missing values *n* (%)
Agree	88 (17.3)	
Partly agree	83 (16.3)	
Neutral	37 (7.3)	
Partly disagree	36 (7.1)	
Disagree	262 (51.4)	
		4 (0.8)
	I have taken more days off *n* (%)	
Agree	47 (9.2)	
Partly agree	78 (15.3)	
Neutral	72 (14.1)	
Partly disagree	47 (9.2)	
Disagree	263 (51.6)	
		3 (0.6)
	I was more absent *n* (%)	
Agree	67 (13.1)	
Partly agree	68 (13.3)	
Neutral	53 (10.4)	
Partly disagree	36 (7.1)	
Disagree	282 (55.3)	
		4 (0.8)
	Due to the COVID-19 pandemic, I consider to reorganize my work *n* (%)	
No, I do not consider reorganizing my work	306 (60.0)	
Yes, I consider working more from home	144 (28.2)	
Yes, I consider quitting working in health care	9 (1.8)	
Yes, I consider working less hours	46 (9.0)	
		5 (1.0)

Missing values are displayed in last column and reflect missed responses on the according question and are calculated using the total sample of respondents (*N* = 510).

With regards to work absenteeism, 16.3% (*n* = 83) to 17.3% (*n* = 88) stated that the (partly agree/agree) had *“taken more sick leave compared to during the pandemic.”* 9.2% (*n* = 47) to 15.3% (*n* = 78) stated they *“took more days off”* and 13.1% (*n* = 67) to 13.3% (*n* = 68) stated they *“were more absent.”* With regards to reorganizing work, 9 respondents (1.8%) were considering *“quitting working in health care,”* 46 (9.0%) were considering *“working less hours,”* 144 (28.2%) were considering *“working more from home”* whereas 306 (60.0%) respondents were *“not considering reorganizing work.”*

### 3.2. Comparison between sample (during and post-pandemic)

Tables 3A,B shows the comparison during pandemic and post-pandemic with regards to the questions about mental health symptoms (Table 3A) and ability to balance work and absenteeism (Table 3B).

**Table 3A:** Comparison of mental health symptoms during pandemic (*N* = 1,372) and post-pandemic (*N* = 510).

I experience…				*Χ*	*p*	*V*
	More symptoms of *n* (%)	Not more/not less symptoms of *n* (%)	Less symptoms of *n* (%)			
*Anxiety*				41.97	<0.001	0.15
During pandemic	262 (19.4)	1,055 (78.3)	31 (2.3)			
Post-pandemic	51 (10.2)	416 (82.9)	35 (7.0)			
*Depression*				27.20	<0.001	0.12
During pandemic	409 (30.3)	911 (67.5)	29 (2.1)			
Post-pandemic	105(21.0)	366 (73.3)	28 (5.6)			
*Higher levels of stress*				24.37	<0.001	0.11
During pandemic	658 (48.5)	650 (47.9)	49 (3.6)			
Post-pandemic	182 (36.0)	294 (58.2)	29 (5.7)			
*Sadness*				13.99	<0.001	0.09
During pandemic	190 (14.2)	1,119 (83.4)	33 (2.5)			
Post-pandemic	49 (9.8)	425 (85.0)	26 (5.2)			
*Anger*				12.94	0.002	0.08
During pandemic	318 (23.6)	994 (73.8)	35 (2.6)			
Post-pandemic	96 (19.1)	379 (75.3)	28 (5.6)			

**Table 3B:** Comparison of ability to balance work and private and questions regarding absenteeism during pandemic (*N* = 1,372) and post-pandemic (*N* = 510).

						*Χ*	*p*	*V*
	Agree *n* (%)	Partly agree *n* (%)	Neutral *n* (%)	Partly disagree *n* (%)	Disagree *n* (%)			
*Due to working from home, work and private balance has tipped the scale towards work*						9.16	0.047	0.07
During pandemic	127 (9.4)	331 (24.6)	387(28.7)	157 (11.7)	345 (25.6)			
Post-pandemic	38 (7.5)	117 (23.2)	123 (24.4)	72 (14.3)	154 (30.6)			
*I can less effectively balance work and private*						17.14	0.002	0.10
During pandemic	213 (15.7)	457 (33.7)	264 (19.5)	126 (9.3)	295 (218)			
Post-pandemic	52 (10.3)	149 (29.5)	107 (21.2)	58 (11.5)	139 (27.5)			
*I cannot balance (anymore) between work and private*						9.27	0.055	0.07
During pandemic	49 (3.6)	224 (16.7)	403 (30.0)	213 (15.8)	455 (33.9)			
Post-pandemic	12 (2.4)	88 (17.4)	122 (24.2)	93 (18.4)	190 (37.6)			
*I have taken more sick leave*						55.57	<0.001	0.17
During pandemic	154 (11.3)	122 (9.0)	109 (8.0)	47 (3.5)	925 (68.2)			
Post-pandemic	88 (17.4)	83 (16.4)	37 (7.3)	36 (7.1)	262 (51.8)			
*I have taken more days off*						29.11	<0.001	0.13
During pandemic	101 (7.5)	168 (12.4)	137 (10.1)	70 (5.2)	875 (64.8)			
Post-pandemic	47 (9.3)	78 (15.4)	72 (14.2)	47 (9.3)	263 (51.9)			
*I was more absent*						39.58	<0.001	0.15
During pandemic	120 (8.8)	106 (7.8)	119 (8.8)	57(4.2)	954 (70.3)			
Post-pandemic	67 (13.2)	68 (13.4)	53 (10.5)	36 (7.1)	282 (55.7)			

With regards to the differences between experiencing mental health symptoms during and post-pandemic (Table 3A), the degree in which mental symptomatology was experienced differed significantly for all symptoms (e.g., anxiety, depression, levels of stress, sadness, and anger). More specifically, levels of anxiety (*Χ*^2^ = 41.97, *p* < 0.001, *V* = 0.15), depression (*Χ*^2^ = 27.2, *p* < 0.001, *V* = 0.12), levels of stress (*Χ*^2^ = 24.37, *p* < 0.001, *V* = 0.11), sadness (*Χ*^2^ = 13.99, *p* < 0.001, *V* = 0.09), and anger (*Χ*^2^ = 12.94, *p* = 0.002, *V* = 0.08) were significantly higher and/or more prevalent during pandemic compared to post-pandemic.

The results in Table 3B show that respondents pointed out that the work/private balance had tipped the scale towards work during the pandemic, more than compared post-pandemic (*Χ*^2^ = 9.16, *p* = 0.047, *V* = 0.07). Furthermore, they were *“less able to effectively balance work and private during the pandemic compared to post-pandemic”* (*Χ*^2^ = 17.14, *p* = 0.002, *V* = 0.10). Moreover, respondents replied that they had taken *“more sick leave post-pandemic compared to during the pandemic”* (*Χ*^2^ = 55.57, *p* < 0.001, *V* = 0.17), had taken more days off (*Χ*^2^ = 29.11, *p* < 0.001, *V* = 0.13), and *“were more absent post-pandemic compared to during the pandemic”* (*Χ*^2^ = 39.58, *p* < 0.001, *V* = 0.15). The amount of respondents that replied to *“not be able to balance (anymore) between work and private”* did not differ significantly during pandemic and post-pandemic.

Table 4 shows the comparison of reorganization of work during pandemic and post-pandemic. Significant differences were found (*Χ*^2^ = 9.06, *p* = 0.029, *V* = 0.07), more specifically a higher amount of respondents replied that they *“were not considering reorganizing their work post-pandemic compared to during the pandemic”* whereas more respondents *“were considering working more from home”* (31.5% vs. 28.5%) or *“were considering quitting working in health care”* (4.2% vs. 1.8%) during the pandemic compared to post-pandemic. Post-pandemic, more respondents (9.1%) *“were considering working less hours”* compared to during the pandemic (7.5%).

**Table 4:** Comparison of reorganization of work during pandemic (*N* = 1,372) and post-pandemic (*N* = 510).

					*Χ*	*p*	*V*
	No, I do not consider reorganizing	Yes, I consider working more from home	Yes, I consider quitting working in health care	Yes, I consider working less hours			
					9.06	0.029	0.07
During pandemic	767 (56.9)	425 (31.5)	56 (4.2)	101 (7.5)			
Post-pandemic	306 (60.6)	144 (28.5)	9 (1.8)	46 (9.1)			

## 4. Discussion

The current study repeated a national-based online survey and reported the prevalence of mental health status of 510 employees in mental healthcare in the Netherlands, post-pandemic. In this way the study created the possibility to compare these outcomes to the former study with a sample of 1,372 respondents during the pandemic, with regard to mental health symptoms. In general, employees stated that their mental symptoms slightly improved after the pandemic. More specifically, the experienced symptoms of mental health complaints were significantly higher during compared to post-pandemic. It became clear that respondents were less able to maintain work/life balance during the pandemic and even reported a shift to work. However, the majority of respondents indicated that they had restored this balance post-pandemic. Moreover, more sick leave was reported post-pandemic than during the pandemic and more frequent absences post-pandemic. As hypothesized, mental symptoms decreased over time. However, the amount or degree of mental health symptoms remains high. Furthermore, the results indicate some sort of resilience with regards to restore of balance between private life and work.

Moreover, the comparison during- and post-pandemic in terms of work reorganization showed significant differences: namely, more respondents indicated that they were no longer considering changing their work compared to during the pandemic. However, more respondents reported working from home or leaving health care during the pandemic than post-pandemic. Respondents indicated that they will work fewer hours post-pandemic compared to during the pandemic.

Our results suggest slight recovery on several fronts. As we found less percentages of symptoms of mental health problems and progression in recovery with respect to work/life balance within the respondents, we became curious about the resilience of the respondents (16). In a review Fletcher et al. (16) stated that “*resilience is constructed in a variety of ways where most definitions are based around two concepts: adversity and positive adaptation. Resilience should have been required in response to different adversities, ranging from ongoing daily hassles to major life events, and that positive adaptation must be conceptually appropriate to the adversity examined in terms of the domains assessed and the stringency of criteria used.”* Moreover, Fletcher wonders whether resilience can be seen as either a trait or a process and conceptualizes resilience as the interactive influence of psychological characteristics within the context of the stress process. There are several studies that suggest that resilience makes people resistant to stress (17). Koelmel et al. (18), stated that with healthcare workers, resilience can be expressed in their possibility to show perseverance and well-functioning self-control by continuously adapting and adjusting to new complex situations and pressure. The higher the level of mental resilience, the more confidence and courage a person has in dealing with difficult situations and circumstances (19). In parallel, if the level of mental health increases, psychological resilience will increase, making people less vulnerable to unexpected or stressful events (20, 21). Resilience refines the relation between perceived risk and potential mental health issues (22). According to Norful et al. (23) and Rieckert et al. (24) better communication, mitigating the stress of Healthcare workers and focusing on improving work- patterns and- conditions supports in building resilience. Preparing young professionals during training how to require resilience in response to some adversities and organizing targeted training on increasing adaptability is recommended. Prior, or during, the development of such training, one should address stigma of being treated by a colleague thus creation of a safe environment, requires clear organizational strategies for mental health status of the staff, consistent and clear communication (10).

This is the first study that explored mental health status longitudinally of Dutch MHCW. The number of respondents was large. One limitation of our sample includes the lack of demographic variables which restrains us in carefully describing the study sample. However, we believe that this anonymity ensured the high response rate in a fairly short amount of time. Furthermore, due the aforementioned limitation, we were unable to explore changes on an individual level. Future studies should continue to focus on the current topic. Furthermore, future studies should incorporate a theoretical framework in order to thoroughly explore adaptability of MHCW with regards to mental health problems. Lastly, we were unable to control for any possible bias (such as early versus late bias, or common method bias) because we did not collect information regarding demographics which we could use to conduct non-response analyses. However, this method did ensure a high(er) response because of the anonymity (e.g., respondents were not required to address their age, gender and so on).

Working in healthcare is stressful, which is stressed by the results of our study. Even though mental symptoms improved after the pandemic, the prevalence rates of depression, anxiety and stress is fairly high. Therefore, institutional meddling to prevent development of these symptoms in for instance burn-out and/or sick leave is pivotal. Especially since we know from the current literature that such feeling are associated with other mental health challenges such as sleep disturbances, occupational impairment, behavioral issues and others. Besides their own responsibility, institutions and governments have responsibility in order to protect employees in healthcare. Especially psychological support is necessary. Promotion of self-care is advised as well as social support (inside and outside of work context). In this sense, providing healthcare to co-workers or colleagues can be part of a psychosocial support program that will increase resilience within an institution. Furthermore, training young professionals to cope with stressful experiences is key and will prevent the development of mental symptoms and ultimately burn-out. The development of such programs needs to be addressed and stimulated.

## 5. Conclusion

This follow-up study, in which a comparison took place between two measurements, a recent measure (October–December 2022, with *N* = 510) versus a previous measure (August–September 2021, *N* = 1,372) referred to as during-pandemic sample and post-pandemic sample, showed a decrease in mental health problems; a better work-life balance; a better work-life ratio; an increase in sick leave and absences post-pandemic and a decrease in people in need of reorganizing work. These results suggest that healthcare professionals are resilient. However, the increase in absenteeism and absence post pandemic is striking.

Implications for policy, practice, and research could be that there will be a change of focus. From combating mental health problems, to prevention and focusing on increasing resilience including the need to carefully manage individuals’ immediate environment, and to search for the protective and promotive factors that individuals can proactively use to build resilience. To prepare healthcare workers and our healthcare system for ongoing demands and possible additional demands such as a next pandemic, research focusing on interventions that will improve mental well -being of healthcare workers and support mental healthcare workers to reduce burnout, sick leave and absenteeism and enhance resilience is necessary. Preparation of the healthcare professionals for future crisis and even a pandemic will contribute to the continuity, availability and quality of health care.

## Data availability statement

The raw data supporting the conclusions of this article will be made available by the authors, without undue reservation.

## Ethics statement

The studies involving human participants were reviewed and approved by CWO GGz Breburg. The patients/participants provided informed consent to participate in this study.

## Author contributions

LV and AB were equally involved in designing the research, data collection, data-analysis, writing drafts, and final editing. All authors contributed to the article and approved the submitted version.

## Conflict of interest

The authors declare that the research was conducted in the absence of any commercial or financial relationships that could be construed as a potential conflict of interest.

## Publisher’s note

All claims expressed in this article are solely those of the authors and do not necessarily represent those of their affiliated organizations, or those of the publisher, the editors and the reviewers. Any product that may be evaluated in this article, or claim that may be made by its manufacturer, is not guaranteed or endorsed by the publisher.
